# Optimization of the production process for the anticancer lead compound illudin M: downstream processing

**DOI:** 10.1186/s12934-022-01886-2

**Published:** 2022-08-17

**Authors:** Lillibeth Chaverra-Muñoz, Theresa Briem, Stephan Hüttel

**Affiliations:** 1 Department of Microbial Drugs, Helmholtz Centre for Infection Research, Brunswick, Germany; 2 German Centre for Infection Research (DZIF), Partner Site Hannover-Braunschweig, Brunswick, Germany

**Keywords:** Solid phase extraction, Crystallization, Liquid–liquid extraction, Natural products, Fungal secondary metabolites, Anti-cancer agents, Small-scale model, Basidiomycota, DSP

## Abstract

**Background:**

Secondary metabolites have played a key role as starting points for drug development programs due to their often unique features compared with synthetically derived molecules. However, limitations related to the discovery and supply of these molecules by biotechnological means led to the retraction of big pharmaceutical companies from this field. The reasons included problems associated with strain culturing, screening, re-discovery, purification and characterization of novel molecules from natural sources. Nevertheless, recent reports have described technical developments that tackle such issues. While many of these reports focus on the identification and characterization of such molecules to enable subsequent chemical synthesis, a biotechnological supply strategy is rarely reported. This may be because production processes usually fall under proprietary research and/or few processes may meet the requirements of a pharmaceutical development campaign. We aimed to bridge this gap for illudin M—a fungal sesquiterpene used for the development of anticancer agents—with the intention to show that biotechnology can be a vital alternative to synthetic processes dealing with small molecules.

**Results:**

We used µL-scale models to develop an adsorption and extraction strategy for illudin M recovery from culture supernatant of *Omphalotus nidiformis* and these findings were successfully transferred into lab-scale. By adsorbing and eluting the product using a fixed resin-bed we reduced the working volume by ~ 90% and removed the aqueous phase from the process. After a washing step, a highly concentrated illudin M fraction was obtained by isocratic elution with 80% methanol. The fraction was dried and extracted using a water/heptane mixture, enriching illudin M in the heptane phase. From heptane illudin M could be instantly crystalized by concentrating the solution, achieving a final purity > 95%.

**Conclusion:**

We have developed a robust, scalable and low-cost downstream process to obtain highly pure illudin M. By using solid phase extraction we reduced the production of solvent waste. Heptane from the final purification step could be recycled. The reduced amounts of solvents required, and the short purification time render this method a very economic and ecologic alternative to published processes.

**Supplementary Information:**

The online version contains supplementary material available at 10.1186/s12934-022-01886-2.

## Background

Cancer is the second most common cause of death worldwide accounting for about 9.6 million deaths in 2018 and for that reason the World Health Organization (WHO) encouraged a global action for the prevention and control of cancer [1]. Natural products (NPs) have contributed remarkably to modern cancer therapeutics and it is estimated that nearly 60% of the current anticancer drugs have been developed, derived or inspired by NPs [2, 3]. Despite this past success big pharmaceutical companies have reduced the efforts to screen natural sources for novel chemical scaffolds; the reasons have been extensively discussed and one is the lack of reliable supplies of these molecules from their natural sources [4].

Illudins M and S are important members of a highly cytotoxic class of fungal sesquiterpenes. Both illudins were originally isolated from the culture broth of *Omphalotus illudens* during a screening for antibiotic substances [5]. The molecules were found due to their activity against *Mycobacterium smegmatis* and *Staphyloccus aureus* but turned out to be highly cytotoxic too [5]. After extensive in vitro and in vivo studies the compounds have been investigated as potential anticancer agents due to their alkylating activity [6–9]. Due to the lack of selectivity of natural illudins towards malignant cells, semisynthetic derivatives with improved selectivity have been developed, for example Irofulven. This is derived from illudin S and is currently in phase II clinical trials for the treatment of castration-resistant metastatic prostate cancer [10]. Furthermore, derivatives based on illudin M have shown improved selectivity in vitro against certain cancer cell lines, but further investigations are needed, which require a sustainable supply of the natural compound [11–13]. We recently reported a stable bioprocess for the production of illudin M achieving high titers [14, 15], however the purification of the product, which is an integrative part of any biotechnological process was required to ensure high yields of pure compound.

Small quantities of illudins have been recovered at laboratory scale either by solid phase or solvent extraction of culture broth [5, 11, 16, 17]. Illudin M has additionally been reported to crystalize after concentration in a hexane/ethyl acetate fraction eluted from silica gel [11], which is desirable since crystalline material can usually be recovered with high purity depending on the crystallization method [18]. At industrial scale, soluble products obtained by submerged cultivation implicate large amounts of aqueous culture broth. This is unfavorable in terms of handling large volumes and problems associated with the removal of water from the process. Therefore, at early stages the product should be concentrated and transferred into organic solvents, which can be removed with less time and energy from the product than aqueous solutions. Two general techniques are commonly applied for removal of water and concentration of small molecules; liquid/liquid extraction with water-immiscible organic solvents and subsequent separation and drying of the organic phase, or solid phase extraction of the product and subsequent elution with organic solvents [19–21].

Due to the large quantities of solvents that would be required for liquid–liquid extractions we focused on the development of a cost efficient solid-phase extraction, which enabled a subsequent chromatographic elution of illudin M. The product-containing fractions were combined, dried and transferred into a crystallization process which enabled an instant precipitation of pure illudin M crystals. Here we report the critical process parameters, which allows for a seamless scale-up of the reported DSP process. This report concludes our work towards the development of a biotechnological process for a stable supply of illudin M to enable medicinal chemistry studies and further pre-clinical and clinical development.

## Results

### Preparation of culture broth

Illudin M is a soluble compound, which does not adhere to the biomass. Therefore, as the primary recovery step for shaken cultures, biomass was removed by centrifugation and the clear supernatant was subsequently filtered through a paper filter using a Buchner funnel. In contrast to the shake flask experiments, bioreactor cultivations [15] were performed using the antifoam SB253 Struktol^®^ (Schill + Seilacher, Germany), which could not be removed from the broth by centrifugation since it formed an emulsion settling as a top layer. Therefore, after centrifugation the supernatant was filtered through a Buchner funnel using a paper filter and a layer of cotton wool. The cotton wool fibers captured the antifoam droplets efficiently and removed the insoluble components completely from the supernatant, which could be directly used for further processing. Yet, stability studies showed that it could be stored in darkness at 4 °C up to 9 weeks without degradation of the product.

**Fig. 1:**
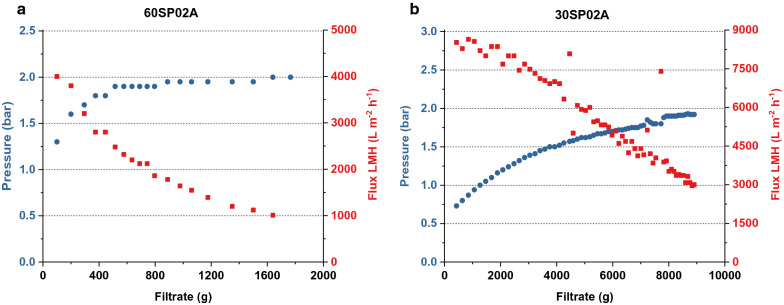
Pressure build-up and flow rate during depth filtration of fermentation broth. Centrifuged culture broth was filtered with comercial depth filters to further reduce particle content. The blue scatter plots indicate the pressure during flitration. The red scatter plots indicate the LMH (Liter per m^2^ per hour) calculated based on filtrate weight and filter area. **a** Pressure and LMH of the run with the 60SP02A filter capsule plotted over weight of the filtrate. The pressure reached 2 bar after about 1.6 L and the flow rate dropped by a factor of 4. **b** Pressure and LMH of the run with the 30SP02A filter capsule plotted over weight of the filtrate. The pressure reached 2 bar after about 9 L while the flow rate dropped by a factor of 3

To avoid damage of sensitive chromatographic equipment a further depth filtration step after antifoam removal was investigated using two different depth filters: 60SP02A (3 M) with a reported nominal retention rating starting at > 0.1 Microns and 30SP02A (3 M) with a retention rating > 0.5 Microns. Both filters were tested using a peristaltic pump, pressure sensors, antifoam-free supernatant and applying a maximal working pressure of 2 bar. Figure 1 shows the pressure increase on the 25 cm^2^ filters plotted over the respective filtration volume. The calculated LMH value (Liter per m^2^ per hour) is plotted in red for better comparison. The data indicated, that the filter 60SP02A (Fig. 1a) allowed a lower throughput and reached the maximum working pressure at < 2000 mL. The filter 30SP02A (Fig. 1b) allowed for higher throughput ~ 9000 mL until it reached the maximum operating pressure.

**Fig. 2:**
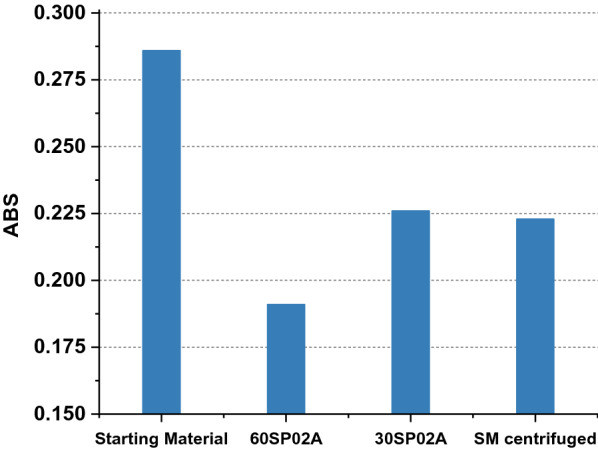
OD600 of different samples to compare efficiency of depth filtration and centrifugation. The bar diagram illustrates the absorbance at 600 nm of samples taken before and after depth filtration with two different filters (30SP02A, 60SP02A). To compare the efficiency of filtration and centrifugation a sample of the starting material was centrifuged for two hours (SM centrifuged). Supernatant filtered with the 60SP02A filter had the lowest absorbance among all samples while centrifugation and filtration with 30SP02A achieved a similar level of clearance. The Y axis does not start at 0.00 for better comparison

The turbidity of the filtrates was compared by measuring the optical density at *λ* = 600 nm (OD600) with a Libra S11 photometer (Biochrom Ltd., UK) and the results are plotted in Fig. 2. The comparison with starting material indicated that the highest reduction in turbidity was achieved with the 60SP02A filter while the 30SP02A filter reduced the turbidity to a value comparable with a sample of the starting material, which was centrifuged at 16,800×*g* for 2 h. None of the filters had an influence on the product concentration.

### Characterization of process parameters in µL-scale

#### Batch adsorption and elution

To investigate the adsorption dynamics of illudin M on XAD16N we added different amounts of the resin (10, 15, 20, 25, 30 and 35 g L^−1^) each into 10 mL of cell-free supernatant from shake flask cultivations containing 293 mg L^−1^ of illudin M. After 30 min of incubation on a shaker at room temperature the illudin M titer in the supernatant was quantified.

**Fig. 3:**
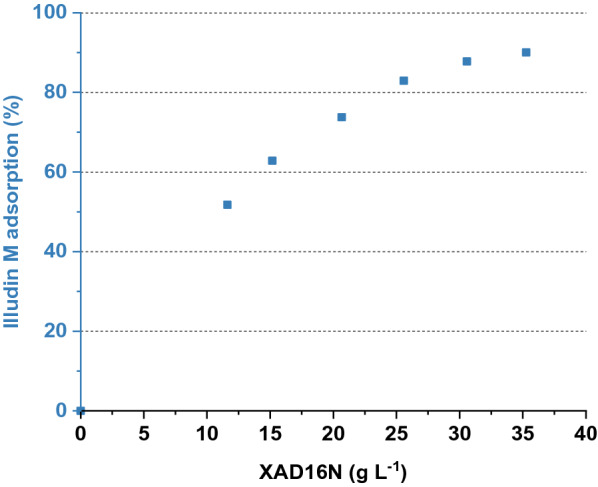
Binding capacity of XAD16N in batch operation. The blue scatter plot illustrates the percentage of illudin M bound to increasing concentrations of XAD16N using 10 mL of cell-free supernatant containing 293 mg L^−1^ of illudin M

Figure 3 shows the percentage of illudin M bound to different concentrations of XAD16N and it was evident that full binding was not achieved under the applied conditions. Changing the pH of the solution (pH 5, pH 7, pH 8) did not influence the binding of illudin M onto the resin.

**Fig. 4:**
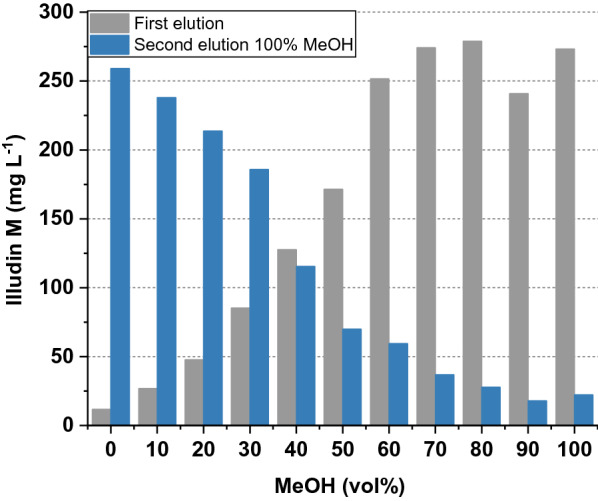
Concentration of Illudin M in extracts of two subsequent elutions from XAD16N incubated with culture supernatant. The gray bars illustrate the illudin M content in the fractions after a first extraction with incresing methanol concentrations. The blue bars illustrate the concentration of illudin M after a second extraction of the resin with 100% methanol. The observed drop at 90% methanol (grey bar) is probably a measurement error since it is not reflected with a higher concentration in the second elution step (blue bar)

For evaluation of illudin M elution, the resin of the previous experiment was pooled, mixed and aliquots of 100 mg were distributed into each of eleven 50 mL tubes. The resin was then incubated on a shaker for 30 min at room temperature with 5 mL of water–methanol mixtures ranging from 0 to 100% of methanol. After this incubation the water–methanol mixtures were removed and a second extraction was performed with 100% methanol under the same conditions. Figure 4 illustrates the resulting illudin M concentration in those eluates. These data indicated that higher methanol concentrations promoted desorption of illudin M from the resin and quantitative elution was achieved at concentrations ≥ 80% methanol.

**Fig. 5:**
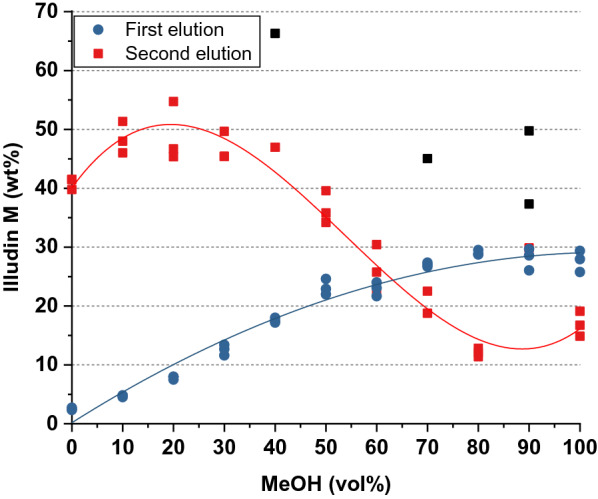
Illudin M concentration indicated in weight percent (wt%) in the dry mass of eluates from two subsequent extraction steps. Blue dots illustrate the wt% of illudin M in the extract from the first elution with increasing methanol concentrations and the respective regression curve. Red squares illustrate the wt% of illudin M in the extract after the second elution with 100% methanol and the respective regression curve. Outliers of the second extraction are illustrated as black squares and may result from measurement errors due to the low weight of the samples. Both data sets were generated with triplicate samples of each eluted fraction

To estimate the weight percentage (wt%) of impurities in the extracts, triplicate samples of 1 mL from each eluate were transferred into weighed vials, dried and the weight of the extract was measured. With the measured illudin M concentration from the previous step and the weight of the extract, the amount of product in each fraction was calculated and correlated to the total amount of extract (see Fig. 5).

The highest proportion of illudin M during the first elution was observed in fractions with > 80% methanol as expected (see Fig. 5). Of greater importance is the observation that during the second elution illudin M reached about 50% purity in the fractions where the resin was previously extracted with methanol concentrations up to 20%. These findings indicated that a considerable amount of undefined components could be removed from the resin by extraction with lower methanol concentrations at the cost of losing minor quantities of illudin M. Based on these observations we decided to test the adsorption and elution dynamics using a column packed with XAD16N as fixed-bed for comparison with the results achieved during the batch adsorption.

#### Fixed-bed adsorption and elution

Two miniature columns with an XAD16N bed of 3.0 mL (1.5 cm bed length) were prepared in order to have a µL-scale model for investigation of adsorption and elution of illudin M. The column was run in a semi-continuous mode by loading 6 mL of clarified supernatant from top and letting it pass through the column. The flow through was controlled by opening or closing a bottom valve.

Since the contact times of product and resin as well as adsorption dynamics are different on a fixed bed compared to a batch adsorption, we investigated the influence of the flow velocity on the adsorption performance. Therefore, we loaded two columns with 35 × 6 mL fractions of supernatant containing 200 mg L^−1^ of illudin M and measuring the product concentration in the flow-through. During the first experiment each fraction ran through the column within ~ 6 s (flow velocity ~ 30 cm min^−1^). In the second experiment the flow-through was slower so each fraction ran through the column within ~ 24 s (flow velocity ~ 7.5 cm min^−1^).

**Fig. 6:**
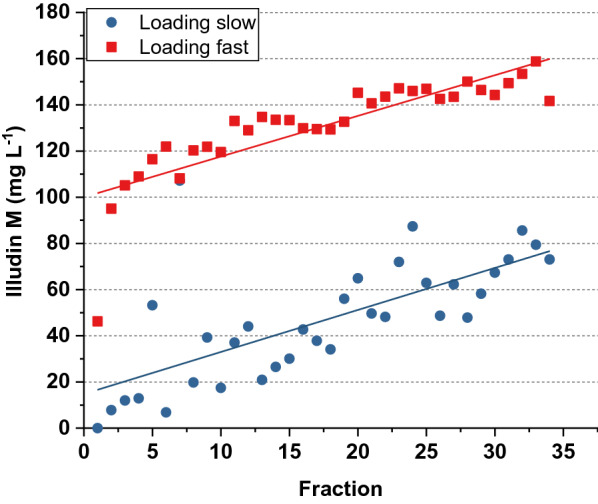
Illudin M concentration in the flow through after loading two XAD16N columns at different flow velocities with culture supernatant containing 200 mg L^−1^ of illudin M. The scatter plots illustrate the illudin M titers in each fraction collected after loading with flow velocity ~ 7.5 cm min^−1^ (blue) and ~ 30 cm min^−1^ (red) with the respective linear fit

The illudin M concentrations measured in each collected fraction are plotted in Fig. 6. The data indicated that a faster flow velocity reduced the binding of illudin M to the packed bed. The graph also illustrates an increase of the illudin M concentration in the flow through over time at both flow rates, which had a linear relationship indicated by the regression lines (see Fig. 6). The results indicated that lower flow rates would be required to obtain an efficient binding of product to the stationary phase.

**Fig. 7:**
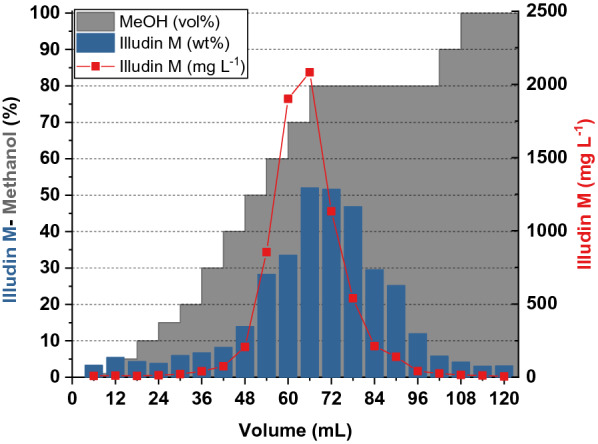
Illudin M concentrations in fractions after stepwise elution of loaded XAD16N column. The red squares (illustrated as a curve to highlight the chromatographic elution) indicate the absolute illudin M concentration in each fraction after extraction from the resin with increasing methanol concentrations. Blue bars illustrate the wt% of illudin M in the total mass of each fraction. The gray area illustrates the percentage of methanol used in each elution step

The resin loaded at higher flow velocity was extracted with 100% methanol, which led to full elution of illudin M within the first four fractions as expected. The resin loaded at lower flow velocity was extracted in a stepwise fashion with increasing methanol concentrations. The elution was done using 6 mL of mobile phase running through the column from top to bottom within ~ 24 s. This resulted in the elution profile illustrated in Fig. 7 in which the concentration of illudin M in the respective fraction is plotted against the elution volume. Since total elution of illudin M was expected–based on the batch experiments—at ~ 80% methanol this elution step was carried out several times (see Fig. 7).The illudin M titer of each fraction was quantified. It was evident that until up to 20% methanol no significant amounts of illudin M were eluted from the column. At higher methanol concentrations illudin M started to elute from the column and at 70–80% a full elution was achieved which tailed over several fractions. By measuring the weight of the fractions and calculating the illudin M content, an enrichment of illudin M to > 50% was observed (see Fig. 7), which confirmed the previous findings from the batch experiments that a considerable amount of impurities could be removed by washing the loaded resin with concentrations < 20% of methanol.

These results indicated that a fixed bed with the XAD16N resin would have better adsorption, enrichment and elution dynamics compared to a batch extraction. Furthermore, the handling of illudin M in a closed chromatography system was considered preferable due to the biological properties of the molecule and a potential upscaling of the developed methods.

### Laboratory-scale studies using culture supernatant from stirred tank bioreactor cultivations

#### Fixed-bed adsorption and elution in a packed column

**Fig. 8:**
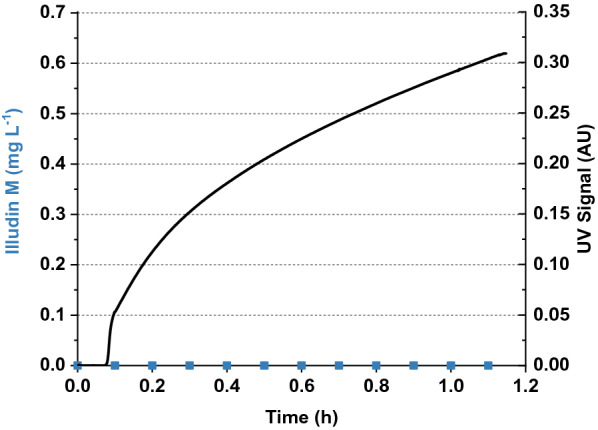
UV signal at 325 nm and illudin M concentration in the flow through during loading experiment. The black curve illustrates the UV signal at 325 nm recorded while loading illudin M on a XAD16N column. Blue squares illustrate the illudin M concentration in samples taken every 6 min from the flow through. The experiment was performed loading 3 L of clear supernatant containing ~ 529 mg L^−1^ of illudin M

The parameters for adsorption and elution established during the µL-scale experiments were used to evaluate the process at laboratory scale. The µL-scale experiments indicated better binding of the compound at lower flow velocities. Therefore the adsorption of illudin M was performed with a linear flow velocity of ~ 5.20 cm min^−1^. Three initial laboratory-scale familiarization runs were conducted. At the flow velocity applied, full binding of the compound was achieved with low back-pressure in the system (< 3 bar). By taking samples every 6 min from the flow-through we ensured that no unbound illudin M passed through the column during loading (see Fig. 8). The UV signal at 325 nm was recorded and showed a steady increase over the loading time.

**Fig. 9:**
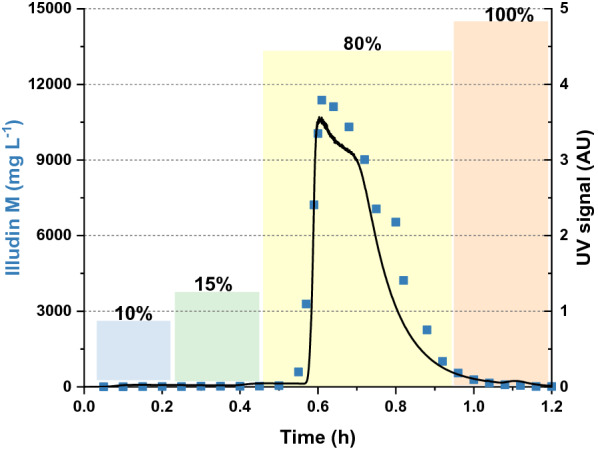
UV signal at 325 nm and illudin M concentration during isocratic elution with different methanol mixtures. The black curve illustrates the UV signal at 325 nm while eluting illudin M from XAD16N with isocratic steps. The blue squares illustrate the illudin M concentrations in samples taken over the run. The colored blocks indicate the methanol concentration and the duration of the isocratic wash and elution steps

The elution was performed with a linear flow velocity of 2.6 cm min^−1^. First we washed the resin with deionized water and then with 10% and 15% methanol. Then the product was eluted with 80% methanol followed by a column-wash step with 100% methanol to regenerate the resin. Fractions were collected every 20 mL. The illudin M titer of every third fraction is plotted in Fig. 9 over the time of elution together with the UV signal at 325 nm, which served as a good indicator to monitor the elution of the product from the resin. The isocratic wash and elution steps are indicated with colored blocks. UV data and titers measured in the fractions indicated the elution of the product as a single peak during the 80% methanol step. The implementation of the washing steps was efficient for enrichment of illudin M (~ 40%) in the eluted fractions. The results were comparable to the data obtained at µL- scale (See Fig. 7 and Fig. 9).

### Quantification of the fixed-bed performance

**Fig. 10:**
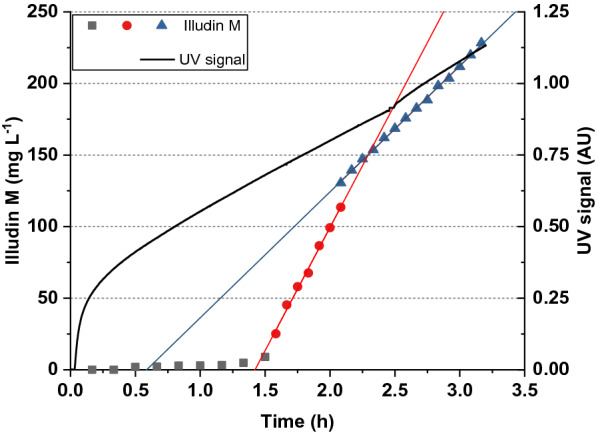
UV signal and illudin M concentration measured while overloading a packed column. A XAD16N column was loaded with 10 L of clarified supernatant (~ 700 mg L^−1^ illudin M). The scatter plot illustrates the concentration of illudin M in the flow through pointing out a breakthrough after 1.5 h (4.5 L). The two different trends of the linear fits are shown in red and blue. The black curve illustrates the UV signal obatined from the chromatography system

Clear filtrate containing ~ 700 mg L^−1^ of illudin M was used to evaluate the loading capacity of a fixed bed. The column was packed with 183 mL of XAD16N and the loading step was performed applying the same conditions as described in the previous section. Samples were taken every 500 mL at the column outlet and once the color of the flow through changed to a darker yellow, samples were taken every 250 mL. The illudin M concentration in those fractions and the UV data are shown in Fig. 10. It is evident that until 4 L had passed through the column (1.5 h), no significant amount of product could be detected in the flow through. From 4.5 L (1.6 h) a breakthrough of product was observed (concurrent with a change in color) followed by a steady increase of the illudin M concentration in the following fractions, which indicated that further loading with these conditions would lead to product loss. Interestingly, the increase in product titer after the flow through was linear but the slope changed after 6.5 L (2.2 h). The UV signal during loading did not indicate a breakthrough of illudin M, thus cannot be used as indicator of product loss.

### Extraction and crystallization experiments to obtain pure illudin M after solid phase extraction

The eluted fractions containing illudin M were pooled in order to perform an extraction of the compound. Since the compound was eluted in 80% methanol from the fixed-bed, we evaluated two different liquid–liquid extraction strategies in µL-scale to determine whether the methanol content would interfere with a subsequent extraction step with heptane.

**Fig. 11:**
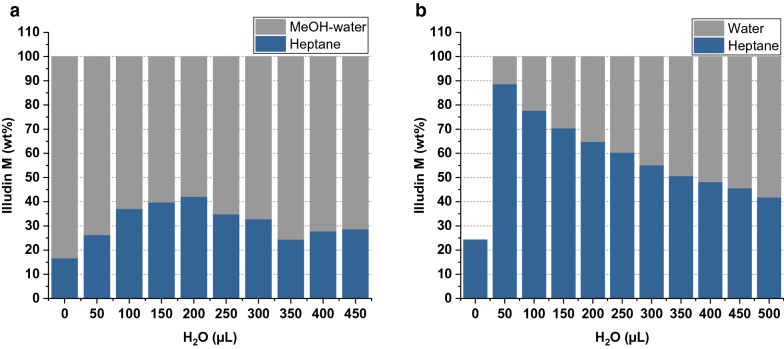
Percentage of illudin M distribution during liquid–liquid extration of eluate with hepane. **a** liquid–liquid extraction of methanol-containing eluates diluted with increased amounts of water and extracted with heptane; the stacked bars illustrate the distribution of illudin M (%) between the methanol/water phase (gray) and the heptane phase (blue). **b** liquid–liquid extraction of dry eluate dissolved in increasing amounts of water and extracted with heptane; the stacked bars illustrate the distribution of illudin M (%) between the aqueous (gray) and the heptane phase (blue)

In the first experiment, 50 µL of methanolic eluate were diluted with increasing amounts of water and extracted with 1000 µL of heptane. To investigate a methanol-free extraction, the second experiment consisted of 50 µL of fully dried eluate, which was resuspended in increasing amounts of water and extracted with 1000 µL of heptane (the dried eluate was not fully soluble in water). To investigate the distribution of illudin M the two phases were separated, dried and dissolved in acetonitrile to quantify the respective illudin M concentration of each. Figure 11 shows the distribution of total illudin M between both phases. It was evident that the presence of even small amounts of methanol kept illudin M in the aqueous phase (see Fig. 11a). When no methanol was present in the aqueous phase (Fig. 11b) the illudin M distribution could be shifted towards heptane by increasing the proportion of heptane to water. A total lack of water prevented efficient dissolution of illudin M in the heptane phase as shown in Fig. 11b in which the dried eluate was extracted only with heptane.

**Fig. 12:**
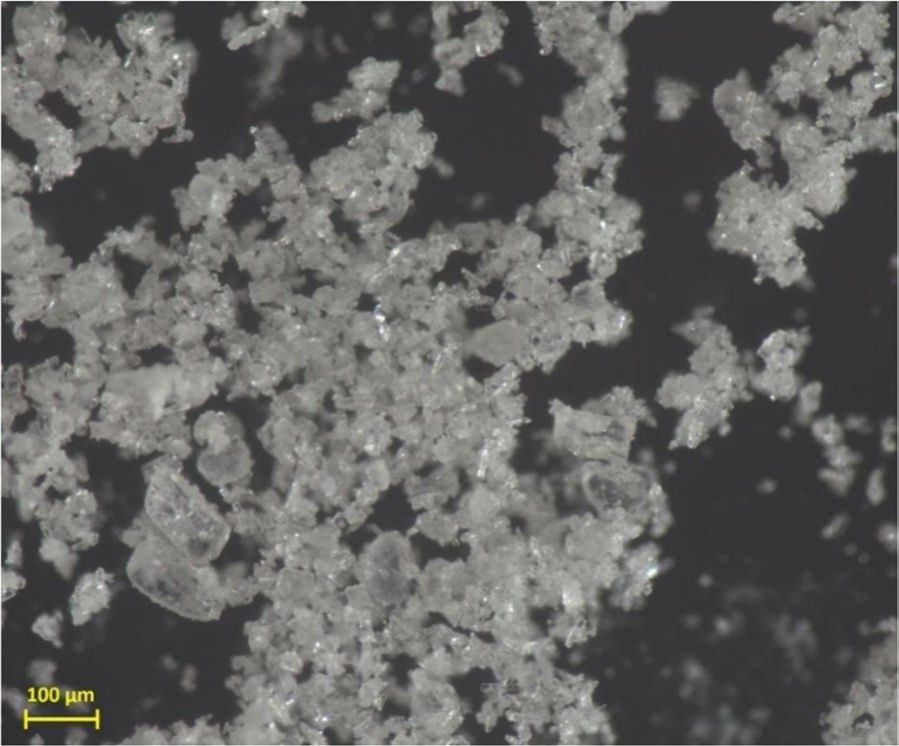
Dried illudin M crystals after concentrating a heptane fraction. A purity > 95% was confirmed using NMR spectroscopy

Based on these data we determined that for upscaled experiments the eluate must be dried (to completely remove methanol) and suspended in water from where illudin M could be extracted with heptane. A ratio of 1:20 of aqueous suspension/heptane was set for the extraction of illudin M based on the results of the µL-scale experiments. With the 1:20 extraction ratio we performed several experiments with larger volumes of eluate in order to test the crystallization of the compound. These experiments indicated that 1 g of dry extract dissolved in 7 mL deionized water was efficiently depleted of illudin M by three extractions with heptane, therefore the 1:7 ratio was set as standard to prepare the aqueous suspension. To perform the extraction the aqueous suspension/heptane mixture was placed in a Schott bottle and stirred on a magnetic stirrer to avoid settling of the suspension and to mix the phases thoroughly. The heptane phase was subsequently recovered by decantation and concentrated by evaporation. Once a high saturation of illudin M in the heptane was reached, crystals started to form and precipitated within a few minutes. Highly pure material was thereby obtained in a very short time (purity > 95%, confirmed by NMR spectroscopy). Figure 12 shows an enlarged picture of dry crystals obtained with this method. The NMR spectra are provided in supplementary information: additional file 1.

### Quantification of overall and crystallization yield

To quantify the overall process yields 3.30 L of culture from a fermentation were centrifuged resulting in 3 L of cell-free supernatant. Removal of biomass reduced the total volume by 10%. The cell-free supernatant was subjected to antifoam removal. In this experiment special care was taken to reduce the height of the cotton layer as much as possible to avoid loss of supernatant and also the subsequent depth filters were completely emptied to obtain maximal yield. Table 1 shows a summary of the calculated yields starting from centrifuged broth after the primary recovery step. The final yield was calculated based on the dry weight of the obtained crystals and was about 77% of the expected illudin M quantified in the cell-free culture supernatant. Sample preparation for process kinetics removed the biomass from the sample and took only the actual titer into consideration. Therefore the volumetric yield of a fermentation—analyzed with this method—must be corrected by a factor of approximately 10% since biomass volume has to be subtracted from the total volume.

**Table 1:** Quantification of overall illudin M yields after each unit operation during DSP

Unit operation	Volume after UO	Titer	Total yield	Yield
(UO)	(L)	(mg mL^−1^)	(g)	(%)
Centrifugation	~ 3.000	0.575	1.725	100
Antifoam removal	~ 3.000	0.575	1.725	100
Depth filtration	~ 3.000	0.575	1.725	100
Adsorption	~ 0.785	1.860	1.460	85
Crystallization	n.a	n.a	1.328	77

In three independent experiments starting from cell free supernatant, final yields of crystals between 72 and 79% could be achieved

### Solubility of illudin M crystals

The solubility of illudin M was tested in several solvents including water. The results are shown in Table 2. Crystals of illudin M were highly soluble in different organic solvents, while poorly soluble in water and heptane.

**Table 2:** Solubility of pure illudin M in different organic solvents and water

Solvent	Solubility (g L^−1^)
Water (deionized)	3.72
Methanol	388.71
Acetonitrile	151.56
Ethyl acetate	182.90
Acetone	271.06
Chloroform	257.28
Dichloromethane (DCM)	205.76
n-Heptane	1.78

## Discussion

We have recently developed a robust biotechnological process delivering high titers of illudin M (> 900 mg L^−1^) in shake flasks [14] and stirred tank bioreactors [15], aiming to supply research grade material for medicinal chemistry studies and further pre-clinical development of improved illudin M derivatives. As a final step to provide pure material for such studies, the isolation of illudin M from complex culture broths was required. Several published reports indicated potential starting points for a scalable downstream process since the compound was shown to bind on a solid phase [5], was shown to be extractable from the aqueous phase with organic solvents [11, 17] and crystals with high purity could be obtained from organic solvents in enriched fractions after a separation over silica gel [11].

We intended to combine these preliminary findings into a simple and scalable method which enables the supply of illudin M with moderate labor and costs. The primary recovery step consisted of biomass removal by centrifugation since the compound was found exclusively in the culture supernatant. For antifoam-containing culture broth, a filtration step with a thin layer of cotton wool turned out to be very efficient to capture antifoam residues that could potentially harm sensitive chromatographic equipment and/or hamper further downstream processing. Subsequent filtration with a set of commercially available filters allowed establishment of scale up parameters and proved to be very efficient when applying pore sizes ≤ 0.5 Micron, since a higher clearance efficiency was achieved compared to centrifugation without any effects on product titers (see Fig. 2). Therefore, a combination of centrifugation and filtration or a sole filtration-based recovery of the supernatant can be considered as primary recovery steps. Applying several filters in serial mode, starting with a high retention rate and reducing pore size in subsequent steps can remove the necessity of centrifugation. This opens the potential to discard single use filters after the clarification of culture broth containing the cytotoxic molecule. Preliminary studies indicated a high stability of the target molecule after removal of biomass when stored at 4 °C in darkness, which eases the handling of the clarified broth and gives flexibility for further processing.

To avoid handling of large volumes of solvents, which are required for liquid/liquid extraction, we investigated the adsorption of the target molecule on a solid phase to remove the aqueous phase from the process, concentrate the product and elute it with a suitable organic solvent. XAD16N is a widely used hydrophobic resin and has been reported in several purification processes for solid phase extraction of target compounds from aqueous phases, displaying high adsorption capabilities in a wide range of operating conditions [22–24]. We investigated the adsorption dynamics of illudin M on XAD16N in batch operation (see Fig. 3) and observed that full binding was not achieved under the applied conditions possibly as a consequence of the low molecular weight (< 250 Da) and the high oxygen content of the molecule (C_15_H_20_O_3_), which shifts the adsorption equilibrium towards the polar aqueous phase. However, by using a µL-scale model a fixed-bed adsorption was evaluated and by determining the influence of the linear flow velocity on product adsorption it was possible to establish a value for this parameter in lab-scale experiments which allows capturing of 1 g of illudin M on 58 mL of resin. In a fixed-bed extraction the adsorbate continuously meets fresh adsorbent in high concentration and is thereby immediately removed during the flow through the column. This allows for a more efficient capturing of the product in comparison to the batch mode, in which a binding-equilibrium occurs between the high volumes of aqueous phase in comparison to the relatively small quantities of solid phase. Full elution of illudin M was achieved with concentrations of methanol ≥ 80% in both, batch operation and fixed-bed mode resulting in comparable outcomes across scales (see Fig. 7 and 9). This rendered fixed-bed adsorption the better alternative to improve recovery efficiency. Handling of the toxin-containing culture broth in a closed chromatographic system increased user-safety and added the advantage of automating and monitoring the process for better characterization.

By investigating the maximum adsorption capacity of XAD16N in a fixed-bed, the experimentally obtained breakthrough point was followed by a slow increase in product concentration over several fractions (see Fig. 6). These results did not match with commonly observed breakthrough curves in which the mass transfer zone (MTZ) or primary sorption zone (PSZ) is relatively narrow, presumably as a consequence of the size of the resin beads [25]. The bead size of XAD16N (20–60 mesh) puts severe restrictions on the achievable resolution. It allows a fast flow around the resin particles at high flow rates resulting in poor penetration of the intra-bead volume and consequently less adsorption of product at the inner bead surface. Increasing adsorption on the bead surface by loading more product, probably reduced the inter-bead volume at a certain point from when the flow was pushed towards the intra-bead volume indicated by the reduced slope at later stages after the breakthrough of product (after ~ 2 h, see Fig. 10). These results indicated that further optimization is possible in either direction: faster loading while reducing the adsorption capacity will reduce process times, while reduced loading speed increases the loading capacity at the cost of longer process times. For the elution step this factor had to be considered as well, since high flow rates would decrease the desorption efficiency. We performed therefore the elution at lab-scale with a lower flow rate than the used for loading the column, which allowed efficient desorption of illudin M reflected by a relatively sharp elution peak.

The increasing UV absorption observed during the loading process (see Fig. 8 and 10) indicated the breakthrough of impurities, which were removed from the process. This effect was further enhanced by washing the fixed-bed with increasing concentrations of methanol up to 15%, which reduced the amount of polar and water-soluble impurities from the cultivation. This led to product concentrations of ~ 40%wt in the fractions eluting at 80% methanol. After the elution the resin could be regenerated with methanol and reused at least two times. Further experiments are required to quantify the performance of the regenerated material.

The last unit operation was the extraction of fractions containing illudin M with heptane for subsequent crystallization. We observed that methanol-containing fractions were not efficiently extracted with heptane (see Fig. 11). This can be explained by the poor solubility of illudin M in either water or heptane but high solubility in methanol (see Table 2, and [5]). Thus removal of methanol from the extract and resuspension in water was necessary to facilitate the extraction with heptane. The removal of methanol enabled the selective extraction of illudin M with heptane since by mixing the dried eluate with water, the water-soluble impurities remained in the aqueous phase while illudin M was extracted into the heptane. The result was a colorless extract from which the product could be instantly precipitated as clear crystals by evaporation of the heptane. The lower solubility of illudin M in heptane compared to water required the use of larger volumes of heptane, yet the organic phase could be completely recovered and recycled after each extraction step. The purity of the obtained crystals was determined by NMR as > 95%, which is sufficient for direct use of the material in following studies.

## Conclusions

In this study we showed that by designing simple µL-scale characterization experiments we were able to investigate the adsorption and desorption dynamics of illudin M on XAD16N. In addition we were able to establish all critical parameters to successfully transfer our findings into a lab-scale purification scheme for rapid illudin M recovery from fermentation broths. The transfer from µL-scale to lab-scale allowed the quantification of the observed effects and yields, which will support further upscaling of this process since all relevant parameters have been identified.

The calculated linear flow velocity of ~ 5.20 cm min^−1^ allowed to capture 1 g of Illudin M on ~ 60 mL of XAD16N. Different titers in the culture supernatant might change the dynamics slightly, but the process has repeatedly shown to be robust under the applied conditions. The presence of antifoam in the submerged fermentations increased the workload in the downstream processing since the latter had to be removed separately after the centrifugation step. Yet, an economical and efficient method was developed to remove it from the culture supernatant. If further clearance of the supernatant is required we propose a depth filtration step to protect sensitive equipment during loading of the compound on the resin. The use of peristaltic pumps for the chromatography allows to skip the depth filtration since the resin itself is not prone to block after the antifoam removal through filtration with cotton and paper filter. The toxicity of the product could be a reason to realize the primary recovery solely based on filtration using single use products.

The reported isolation of gram amounts of pure illudin M crystals from fermentation broth is a fast process, which has been carried out within less than 10 h. These findings in combination with the reported production process are the base of a scalable, economical and efficient biotechnological process, which will enable a reliable illudin M supply and support further work with this interesting molecule.

## Methods

### Miniature-scale packed column

To resemble a small column we used a 10 mL syringe (Braun, Germany) without piston. A layer of gauze prevented the resin from being purged out of the syringe. A layer of 3 mL of XAD16N resin (~ 1.5 cm bed length) was covered with gauze and one layer of sea sand. The miniature column was loaded stepwise by pipetting 6 mL from top of the column. The flow was controlled by opening and closing a clamp on a silicon hose which was connected to the outlet of the syringe. Two different setups were tested to evaluate different loading and elution flow rates. In the first set up the tubing connected to the column had a 4 mm ID and an on–off clamp to allow fast flow through. The second was built with a 2 mm ID tubing and an adjustable clamp to control slower flow rates.

### Primary separation and antifoam removal

Illudin M was produced by submerged fermentation in stirred tank bioreactors [15]. The culture broth was separated from the biomass by centrifugation at 15,000×*g* for 30 min using the Sorvall RC-5B Refrigerated Superspeed Centrifuge (DuPont Instruments) at room temperature using the fixed-angle rotor Fiberlite™ F9-4 × 1000y (Thermo Fischer, USA). After centrifugation, a layer of antifoam formed on the surface of the supernatant which could not be fully removed. To eliminate antifoam residues, the supernatant was vacuum filtered using a Büchner funnel with a double layer of ø150 mm paper filter MN 615 (Macherey–Nagel) and an additional 4 cm layer of cotton wool on top. This setup was sufficient to clear > 10 L supernatant from antifoam without clogging and no significant drop in performance. The filtrate was collected in glass bottles and stored at 4 °C in darkness until further usage.

### Depth filtration

Depth filtration of antifoam-free supernatant was done with Zeta Plus™ filter capsules (3 M, USA) using a SARTOFLOW^®^ Benchtop Crossflow System with the Tandem 1082 Peristaltic-Pump, respective pressure sensors (0 to 5 bar) and the laboratory balance Talent TE 4101 (Sartorius, Germany). The filters were purged with ultrapure deionized water according to the manufacturer’s instructions, air was removed from the filter-housing and the filters were subsequently used for filtering from top to bottom placing the pressure sensor between pump and filter. The flow was set to 100 mL min^−1^ for the BC0025S60SP02A capsule and 200 mL min^−1^ for the BC0025S30SP02A capsule (3 M, USA). The maximum working pressure was set to 2 bar. The filtrate weight and pressure were recorded manually.

### Solid phase adsorption and elution

Clarified supernatant was pumped through a fixed bed of Amberlite^®^ XAD16N resin using a MPLC C-600 system with two pumps and a C-35 UV photometer (Büchi Labortechnik GmbH). Pressure and UV-data at 325 nm were recorded using the Sepacore^®^ software from Büchi. The resin was packed in a TAC35/250SLG0-SR-2 column (YMC GmbH) with 35 mm ID and equipped with solvent resistant FEP tubing (1.6 mm ID and 3.2 mm OD).

For loading, the supernatant was pumped through the column from bottom to top to avoid trapping of air bubbles. The resin bed had a length of 190 mm (183 mL column volume) and the pump rate was set to 50 mL min^−1^, which equates a flow velocity of 5.19 cm min^−1^. After loading, the column was washed with five column volumes of ultrapure degassed water at the same flow velocity. Then, a stepwise elution was performed with methanol/water mixtures at a flow velocity of 2.6 cm min^−1^. The volume and composition of each elution step are listed in Table 3. Samples were taken every 250 mL during runs to quantify illudin M concentrations in the flow through. Fractions of 15 mL were collected automatically in 20 mL glass tubes.

**Table 3:** Methanol concentrations during stepwise elution and colume volumes used per each step

Methanol	Run volume (cv)
0%	5.0
10%	2.7
15%	1.4
80%	4.8
100%	1.4

### Liquid–liquid extraction and crystallization

Eluted fractions containing illudin M were pooled together in a round bottom flask for subsequent compound concentration by evaporation using a Hei-VAP Precision rotary evaporator (Heidolph GmbH, Germany). The heating bath was set to 40 °C, the rotation was 150 min^−1^ and the pressure was slowly reduced to 50 mbar to avoid boiling. The process was finished when the methanol/water mixture was fully evaporated.

The dry eluate was re-suspended in ultrapure water at a ratio of 1:7 (eluate/water) and sonicated in a water bath for about 3 min to obtain an aqueous suspension that was subsequently extracted with heptane at a ratio of 1:20 (suspension/heptane). The mixture was stirred at room temperature in a 1 L Schott flask on a magnetic stirrer at 800 min^−1^ for 30 min. The heptane phase was recovered using a separating funnel and placed in a 1 L round bottom flask. The heptane was removed with the rotary evaporator and recycled. The aqueous suspension was extracted three times with heptane. Crystals started to appear in the round bottom flask during the evaporation of heptane. At this stage the round bottom flask was placed in an ultrasonic bath for three minutes and illudin crystals precipitated as a white powder. The remaining heptane was collected in a fresh 1 L round bottom flask and combined with the next extraction step. The crystals were washed with ice-cold heptane, collected in a 50 mL tip bottom flask and subsequently dried under high-vacuum.

### Analytical methods

The procedure for product quantification and MS analysis have been previously described [14]. However, a summary of the descriptions is presented in this section for the convenience of the reader.

### Product quantification

Samples of 200 µL of supernatant were mixed 1:1 with ice-cold acetonitrile and centrifuged for five min at 16,800×*g*. The illudin M concentration was determined via RP-HPLC analysis using 100 µL of the prepared sample but injecting 4 µL for measurement. The system used for the measurements was the Dionex UltiMate™ 3000 UHPLC System (Thermo Fischer Scientific™) equipped with a DAD detector on an Acquity UPLC^®^ BEH C18 colum (1.7 μm, 2.1 mm × 50 mm, Waters™), at 40 °C. The mobile phase consisted of (A) H_2_O + 0.1% formic acid (FA) and (B) acetonitrile + 0.1% FA at a flow rate of 600 μL min^−1^.

Separation of the sample was achieved by a linear gradient initiated by a 0.5 min isocratic step at 5% (B) followed by an increase of (B) to 40% until 7.4 min. Subsequently the column was cleaned for the next injection by a linear increase to 100% (B) until 8.5 min, and an isocratic step at 100% (B) until 10.5 followed by a linear decrease to 5% (B) until 11.0 min, and equilibration with an isocratic step at 5% (B) until 13.0 min. The UV signal for quantification was set to 325 ± 10 nm. The calibration curve was established using a sample of the pure compound (> 95% purity).

### High resolution mass spectrometry (HR-MS)

High resolution electrospray ionization mass spectrometry (HRESIMS) was performed with an Agilent 1200 series HPLC–UV system combined with an ESI-TOF–MS (maXis^®^, Bruker) applying the analytical conditions described in the section for product quantification. Illudin M showed a molecular ion in the positive Electrospray mode at m/z 271.1305 [M + Na]^+^ (calculated for C_15_H_20_NaO_3_^+^, 271.1307).

### NMR spectroscopy

A Bruker Avance III 500 MHz spectrometer equipped with a BBFO (plus) SmartProbe (^1^H 500 MHz, ^13^C 126 MHz) was used to confirm the structure and purity of illudin M crystals by measuring ^1^H, ^13^C, COSY, HSQC, HMBC of the molecule in CDCl_3_ and confirming chemical shifts with reported values.

### Determination of solubility

The solubility of the crystals was evaluated in different solvents including deionized water, n-heptane, methanol, acetonitrile, chloroform, dichloromethane (DCM), ethyl acetate and acetone. Samples were prepared adding small amounts of the compound into 1000 µL of each solvent until saturation. The mixtures were shaken for 15 min at 1000 min^−1^ with a ThermoMixer^®^ C (Eppendorf). Tubes were afterwards centrifuged at 16,800×*g* for 10 min and 50 µL aliquots were taken for quantification by RP-HPLC. Samples from n-heptane, chloroform, DCM, and ethyl acetate were dried and solved in acetonitrile prior to quantification.

## Supplementary Information

**Additional file 1**: **Table S1.** Chemical shifts of Illudin M in CDCl3 (reported* and measured data). **Figure S1.** Enlargement of non-illudin M protons in the 1H spectrum of illudin M crystals. **Figure S2.** Proton NMR spectrum. **Figure S3.** Carbon NMR spectrum. **Figure S4.** COSY spectrum. **Figure S5.** HSQC spectrum. **Figure S6.** HMBC spectrum.

## Data Availability

All datasets generated during this study can be available from the corresponding author upon reasonable request.

## Abbreviations

DSP: Downstream processing
NPs: Natural products
RP-HPLC: Reverse phase high performance liquid chromatography
HRMS: High-resolution mass spectrometry
NMR: Nuclear magnetic resonance
COSY: Correlation spectrometry
HSQC: Heteronuclear single-quantum coherence
HMBC: Heteronuclear multiple-bond correlation
BBFO: Broadband fluorine observe
DMSO: Dimethyl sulfoxide
DCM: Dichloromethane
CDCl_3_: Deuterated chloroform
