# Supplementary material for: Optimization of the production process for the anticancer lead compound illudin M: downstream processing
Source: Microb Cell Fact. 2022 Aug 17;21:165. doi: 10.1186/s12934-022-01886-2 (PMC9382783; doi:10.1186/s12934-022-01886-2)
Supplement: Supplementary file 1 — Additional file 1: Table S1. Chemical shifts of Illudin M in CDCl3 (reported* and measured data). Figure S1. Enlargement of non-illudin M protons in the 1H spectrum of illudin M crystals. Figure S2. Proton NMR spectrum. Figure S3. Carbon NMR spectrum. Figure S4. COSY spectrum. Figure S5. HSQC spectrum. Figure S6. HMBC spectrum. [file 12934_2022_1886_MOESM1_ESM.pdf]

Additional file 1

Optimization of the production process for the anticancer lead compound illudin M: downstream processing

Lillibeth Chaverra-Muñoz[1, 2], Theresa Briem[1], Stephan Hüttel*[1, 2]

1. Department of Microbial Drugs, Helmholtz Centre for Infection Research, Brunswick, Germany

2. German Centre for Infection Research (DZIF), Partner Site Hannover-Braunschweig, Brunswick, Germany.

1. Comparison of analytical data from purified illudin M with published data

Table S 1 Chemical shifts of Illudin M in $CDCl_3$ (reported* and measured data)

**Literature 1H**	**Measured 1H**	**Literature 13C**	**Measured 13C**
0.43 (m, 1H)	0.40 (m, 1H)	200.5	200.6
0.83 (m, 1H)	0.83 (M, 1H)	146.6	146.8
0.96 (m, 1H)	0.94 (m, 1H)	138.8	138.9
1.11 (s, 3H)	1.09 (s, 3H)	134.5	134.6
1.15 (m, 1H)	1.11 (m, 1H)	132.9	133.1
1.17 (s, 3H)	1.19 (s, 3H)	78.9	79.0
1.37 (s, 3H)	1.35 (s, 3H)	75.9	76.1
1.55 (br s, 1H)	1.54 (br s, 1H)	49.1	49.2
1.69 (s, 3H)	1.67 (s, 3H)	31.6	31.8
3.55 (br s, 1H)	3.57 (br s, 1H)	27.3	27.4
4.41 (s 1H)	4.39 (s, 1H)	24.8	24.9
6.54 (s, 1H)	6.53 (s, 1H)	20.5	20.6
		14.2	14.3
		not reported	8.8
		not reported	6.1

*F.R. Kinder, K.W. Bair: **Total synthesis of (±) Illudin M**. J. Org. Chem. 1994, 59, 6965-6967

$C_{15}H_{21}O_3$: m/z $[M+Na]^+$ calculated 271.1305, m/z $[M+Na]^+$ measured 271.1307

m/z $[M-OH]^+$ calculated 231.1385, m/z $[M-OH]^+$ measured 231.1377

2. Estimation of purity using 1H NMR data

To verify the purity of the crystalline material. A sample of 10 mg was dissolved in 500 µl $CDCl_3$ and a proton spectrum was recorded. The integration of the most dominant impurities in the spectrum (highlighted in Fig. S 1) indicates that the proportion non-illudin M to illudin M protons is smaller than 5%.

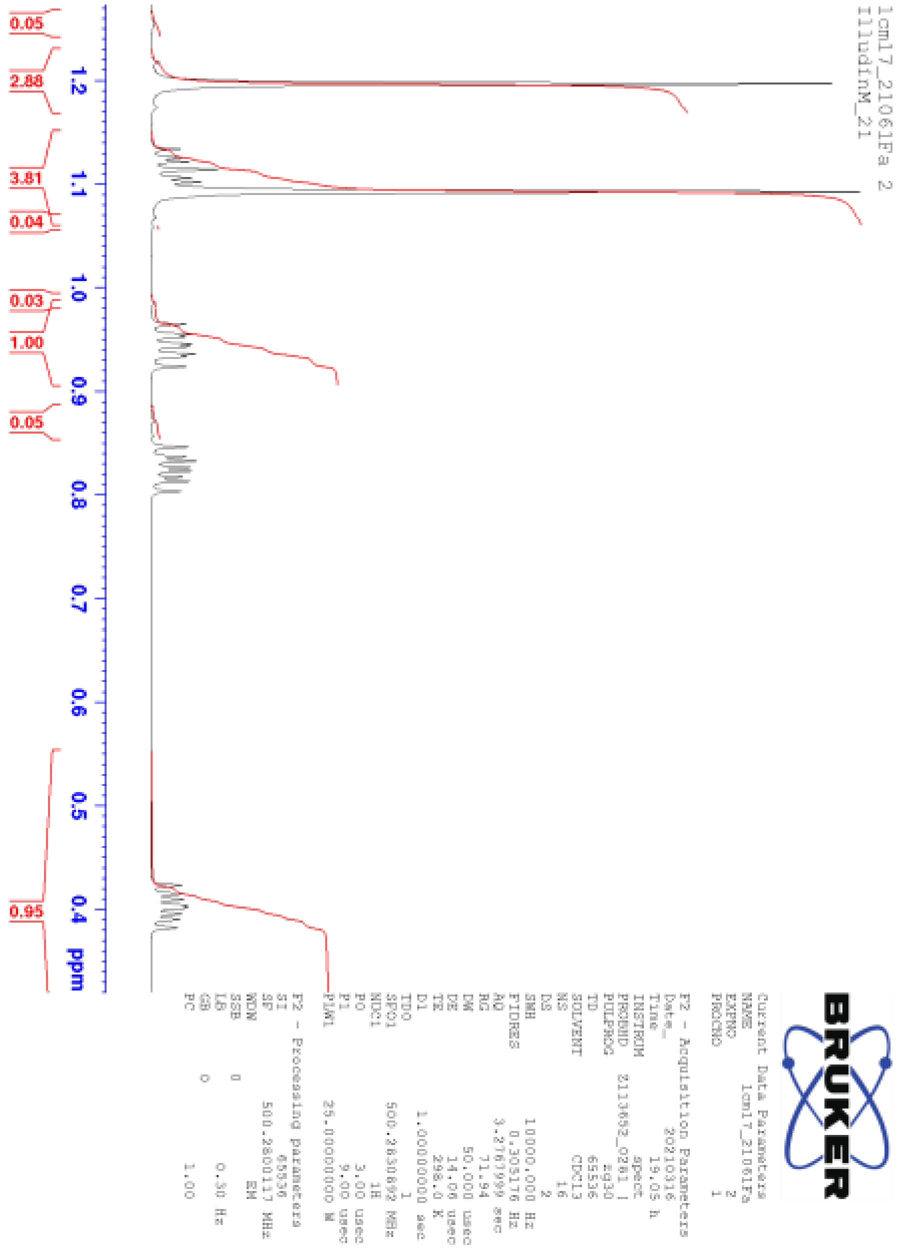

Fig. S 1 Enlargement of non-illudin M protons in the 1H spectrum of illudin M crystals.

3. NMR spectra

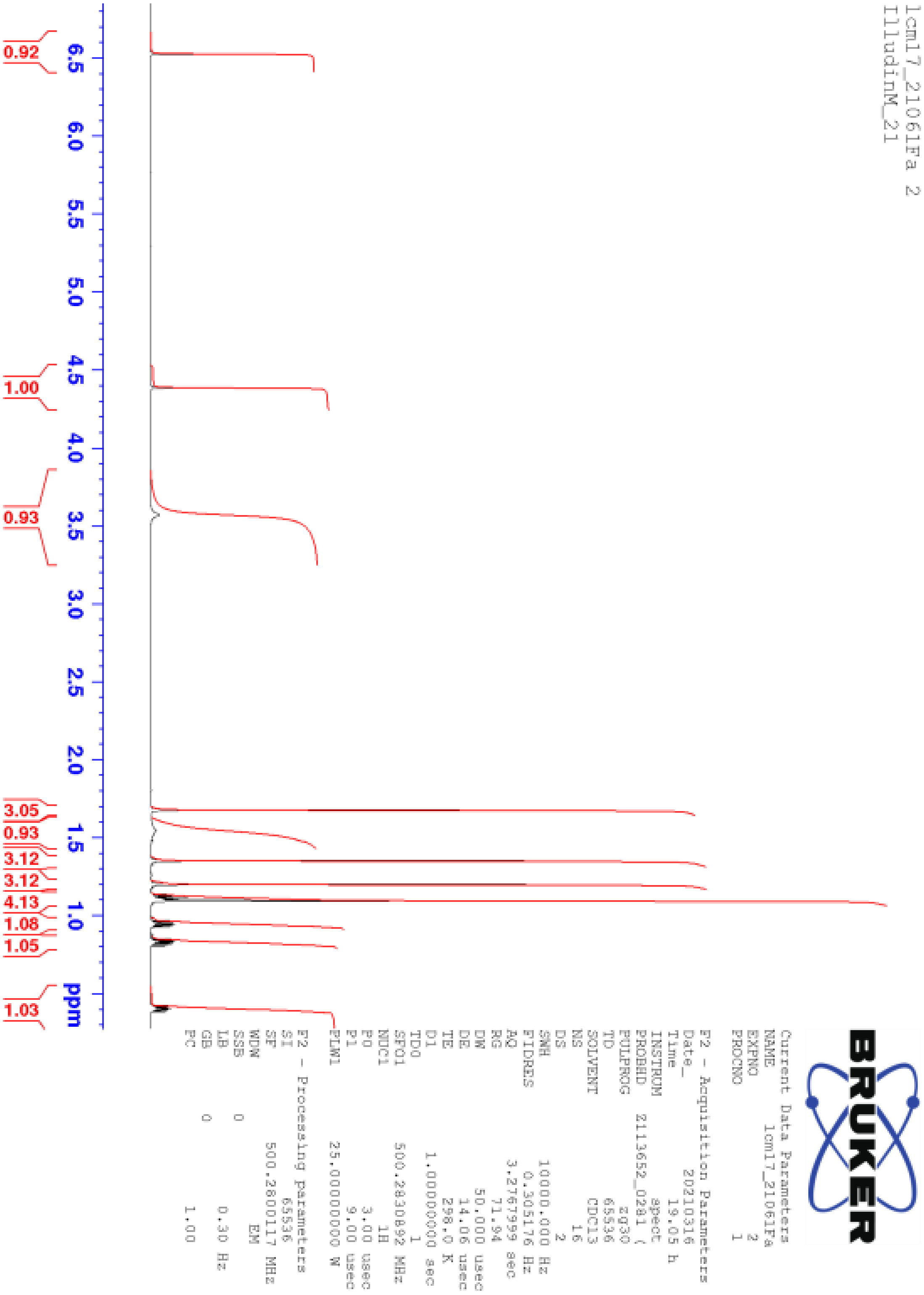

Fig. S 2 Proton NMR spectrum

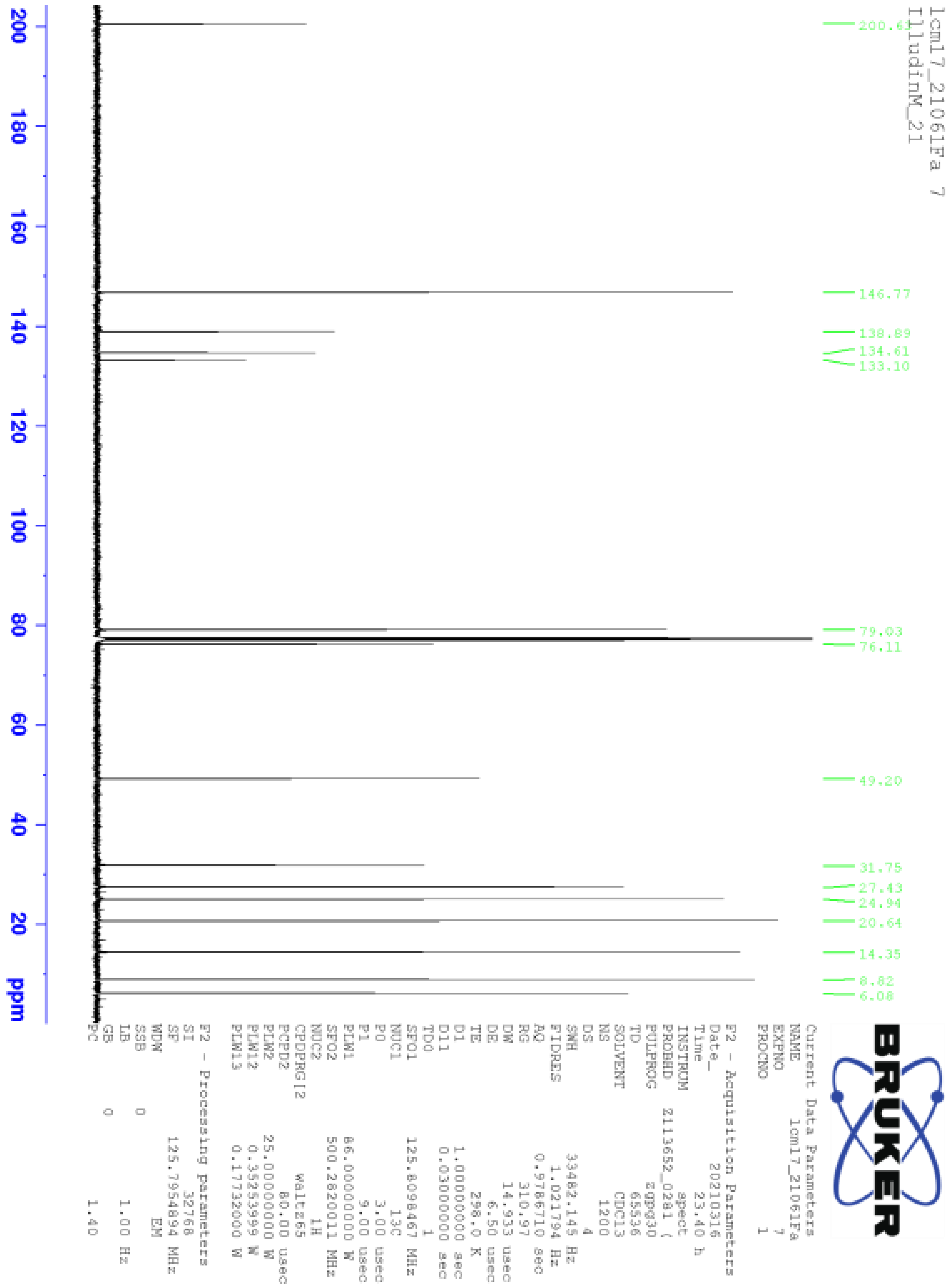

Fig. S 3 Carbon NMR spectrum

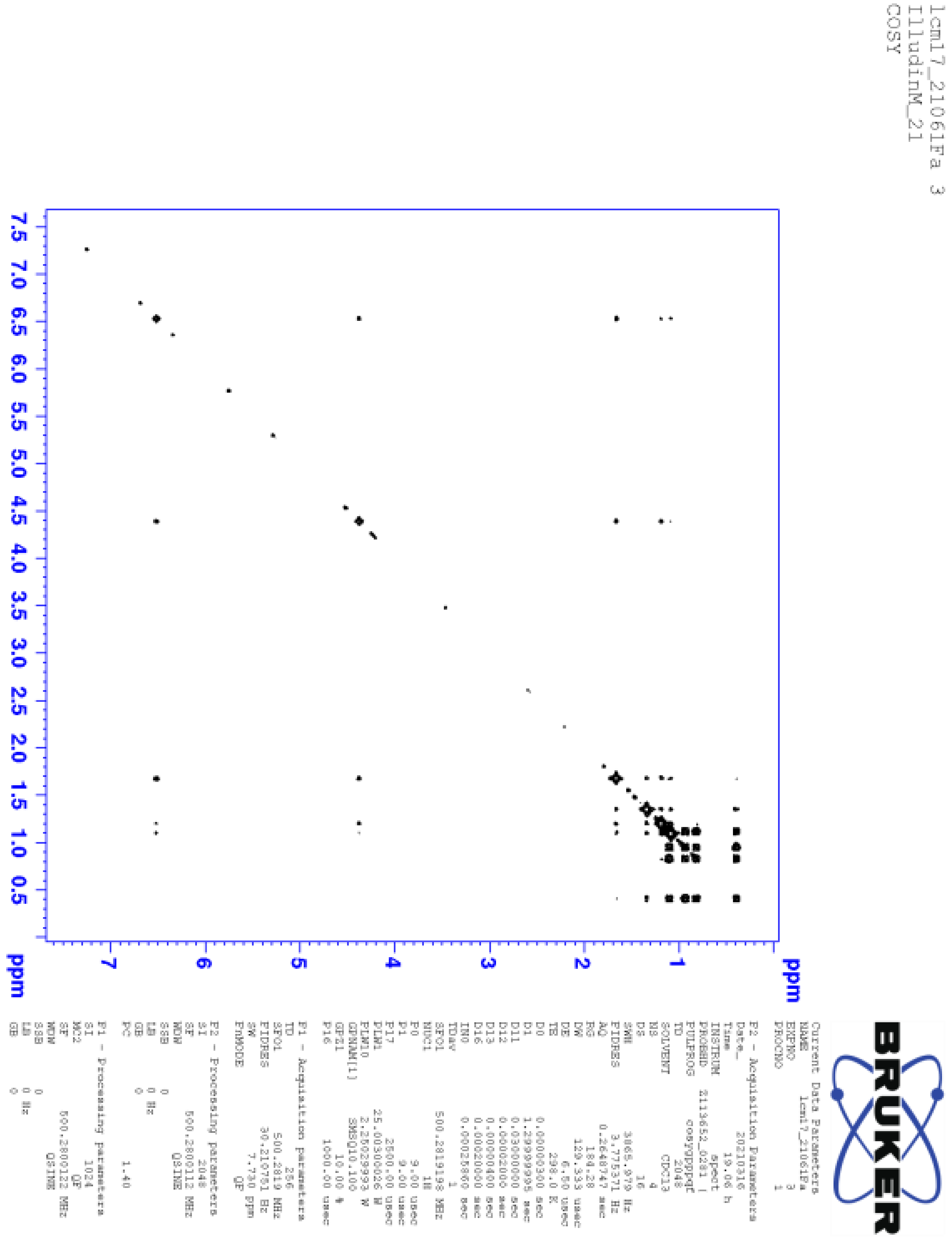

Fig. S 4 COSY spectrum

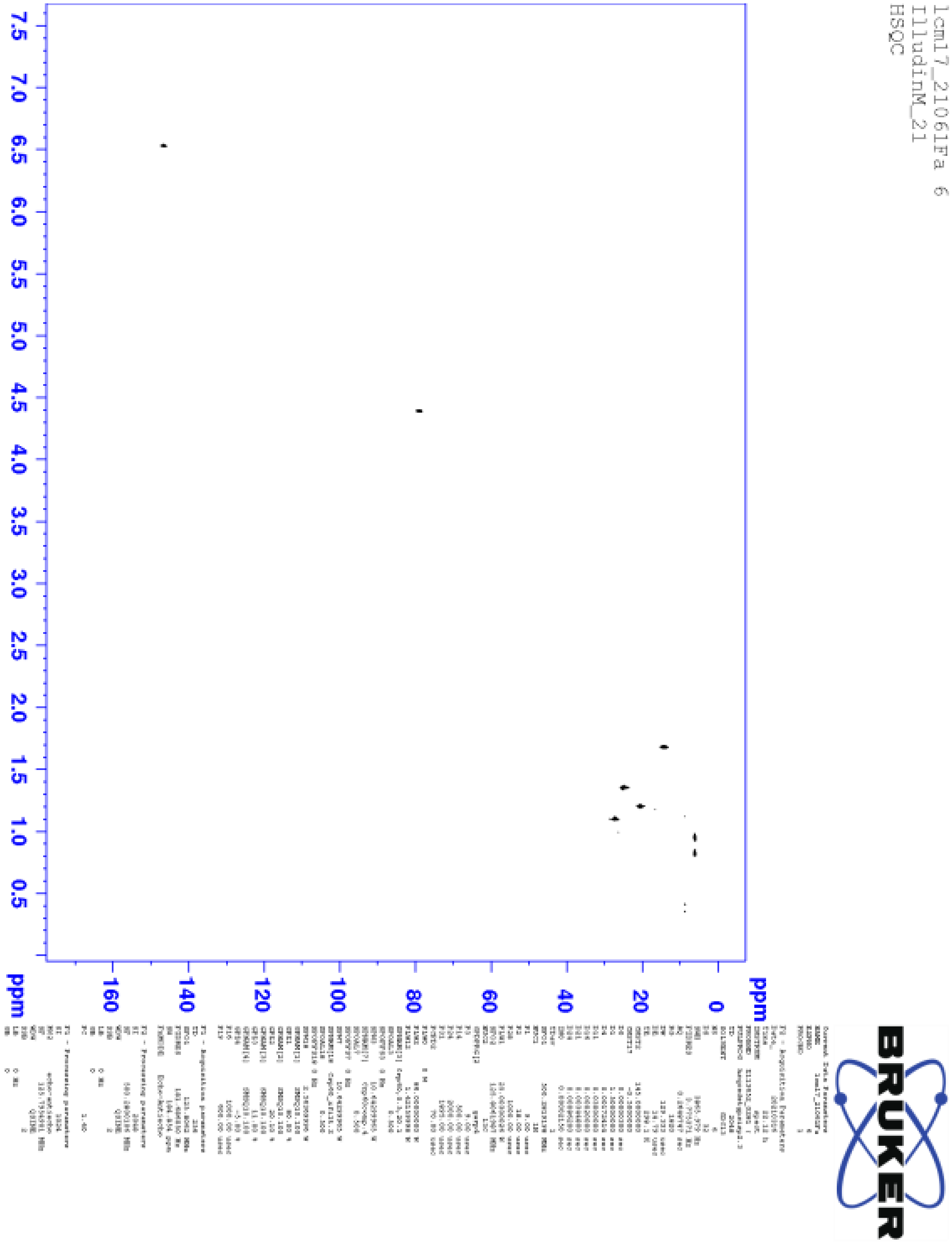

Fig. S 5 HSQC spectrum

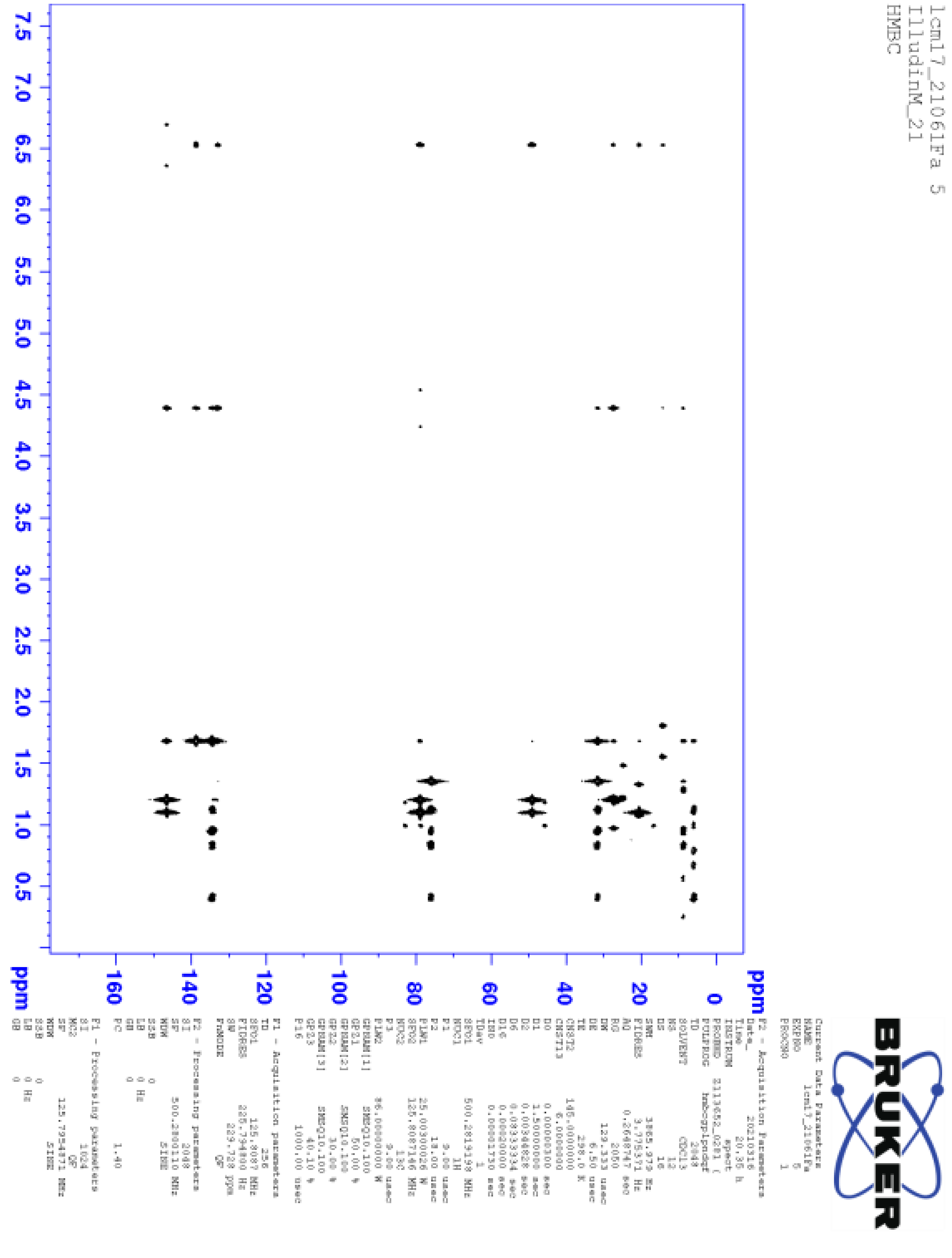

Fig. S 6 HMBC spectrum
